# Palliative Care Challenges of Patients With Progressive Bulbar Palsy: A Retrospective Case Series of 14 Patients

**DOI:** 10.3389/fneur.2021.700103

**Published:** 2021-09-24

**Authors:** Sarah K. Bublitz, Christiane Weck, Andrea Egger-Rainer, Katharina Lex, Piret Paal, Stefan Lorenzl

**Affiliations:** ^1^Palliative Care Research Hub at the Institute of Nursing Science and Practice, Paracelsus Medical University, Salzburg, Austria; ^2^Department of Neurology, Agatharied Hospital, Hausham, Germany

**Keywords:** amyotrophic lateral sclerosis, progressive bulbar palsy, palliative care, oral secretion management, end-of-life

## Abstract

Progressive bulbar palsy (PBP) is a form of motoneuron disease and is widely classified as a subtype of amyotrophic lateral sclerosis (ALS) with a shorter time of survival and female predominance. In this retrospective case series of 14 patients with PBP, we focus on challenges in palliative care for this patient cohort, including symptom control, gastrostomy, non-invasive ventilation, and end-of-life phase. We show that rapid physical decline at the end of life is associated with bronchopulmonary infection and excessive oral secretion leading to a high level of symptom burden. Early and regular advance care planning discussions with a focus on oral secretion management with patients and caregivers are crucial.

## Introduction

Early integration of palliative care is an important task when treating patients with amyotrophic lateral sclerosis (ALS) (1, 2). ALS is a progressive neurodegenerative disease with a high symptom burden, and in the majority of cases, ALS leads to paralysis of the limbs, impaired speech, swallowing, and ventilatory failure. The rapid progression of the disease and the question of whether or not to include life-prolonging therapies, such as feeding tube placement or ventilatory support, should be a stimulus for early discussion regarding therapeutic limitations and advance care planning (ACP).

ALS and motoneuron disease (MND) are generic terms for a range of different phenotypes defined by a varying involvement of spinal and bulbar upper and lower motoneurons (3). Progressive bulbar palsy (PBP), or bulbar phenotype, is defined as bulbar onset with dysarthria and/or dysphagia, tongue wasting, fasciculations, and no peripheral spinal cord involvement for the first 6 months after symptoms onset (3). The median survival time in bulbar phenotype patients is shorter than in other subgroups (3, 4).

An analysis of a large Italian ALS population found a correlation of the bulbar phenotype with older age and women being more affected than men (5). Expansions in the gene *C9orf72* were related to a significant increase of the bulbar phenotype, and patients with bulbar phenotype had an increased risk of developing cognitive impairment and were more likely to develop frontotemporal dementia (FTD) (5). Recent diffusion tensor imaging (DTI) data showed the same microstructural involvement in both PBP and ALS, supporting the hypothesis of a phenotypic spectrum of the same disease (6). However, the discussion of whether PBP might be seen as a subgroup within a spectrum of ALS or as a distinct entity continues (7).

This paper presents a retrospective case series of PBP patients in palliative care, including symptom control, course of the disease, and end-of-life phase in a relatively homogenous group of patients.

## Methods

This is a retrospective case series of patients with PBP in palliative care, which was conducted from September 2017 till January 2021. During this time, 91 ALS patients were treated at our hospital as in-patients and out-patients. Patient records were analyzed regarding demographic and clinical data, medication, course of the disease, and end-of-life phase.

PBP patients were identified according to the diagnostic criteria for PBP (3, 8). All patients (*N* = 14) showed isolated bulbar onset with dysarthria and/or dysphagia, tongue wasting, fasciculations, and no peripheral spinal involvement for the first 6 months after symptom onset. Of the 14 patients, 1 was male. Four patients are still alive; one is currently on invasive ventilation *via* tracheostomy.

## Results

Demographic data of the 14 PBP patients are shown in Tables 1A,B. The median age at the time of symptom onset was 68.5 years. The time between symptom onset and diagnosis varied between 3 and 16 months. Ten out of the 14 patients have died, with a median survival time from symptom onset until death of 27.5 months. In 11 patients, PBP manifested with isolated dysarthria, 1 patient (No. 4) noticed swallowing problems and burning of the tongue as the first symptom, and 2 patients (Nos. 3 and 13) reported dysarthria and dysphagia as having occurred together. Eight patients developed weight loss in an early phase of the disease, within the first 6 months: Nos. 1, 2, 3, 7, 9, 10, and 13.

**Table 1A:** Demographic and clinical data of patients with progressive bulbar palsy.

**Patient** **no**.	**Age at onset** **(years as range)**	**Time symptom onset** **to diagnosis** **(months)**	**First symptom**	**Significant early** **weight loss**	**Time onset to** **first spinal** **symptoms (months)**	**PEG placement** **(time from onset) (months)**	**NIV initiation** **(time from onset)** **(months)**	**Treatment efforts** **against sialorrhea**	**Mechanical insufflator/** **exsufflator device** **initiated**	**Death** **(time from onset)** **(months)**
1	60–65	12	Dysarthria	Yes	30	24	24 Not well-tolerated and rarely used due to sialorrhea	Scopolamine had little effect, Mestinon no effect, Amitriptyline resulted in unpleasantly dry mouth. Botulinum toxine mediocre effect on drooling, thick mucus persistent.	Yes	31
2	50–55	4	Dysarthria	Yes	23	21	23 Not well-tolerated due to siallorrhea	Pirenzepine and Scopolamine showed no effect	Yes	29
3	70–75	15	Dysarthria + dysphagia	Yes	18	16	30 Initiated on ICU 1 week prior to death	Amitriptyline	No	30
4	80–85	6	Burning of tongue, dysphagia	No	12	21	21 Not tolerated when tested due to constriction of pharyngeal muscles on exspiration	Scopolamine lead to cognitive alteration, Amitriptyline to daytime tiredness	No	27
5	56–60	10	Dysarthria	No	30	19	16 Regular use max. 3–4 h/night, in the second half of the night severe dryness of the mouth	Amitriptyline lead to circulatory problems. Scopolamine showed mediocre effect on drooling.	No	TIV
6	70–75	12	Dysarthria	No	24	22	22 Not tolerated when tested due to constriction of pharyngeal muscles on exspiration	Scopolamine showed little effect on drooling, thick mucus persistent. Atropine lead to cognitive alteration. Botulinum toxine mediocre effect. Radiotherapy not feasible due to orthopnoea	Yes, but not tolerated	37
7	70–75	11	Dysarthria	Yes	28	No PEG	No indication	Scopolamine and Amitriptyline discussed several times, patient decided against medication	No	28
8	66–70	3	Dysarthria	No	23	17	Decided against NIV	Scopolamine initially with good effect, later severe skin irritation. Amitriptyline mediocre effect. Botulinum toxine and Radiotherapy without satisfying effect.	Yes, but not tolerated	25
9	66–70	10	Dysarthria	Yes	17	14	No indication	Scopolamine initially effective	Yes	20
10	66–70	16	Dysarthria	Yes	24	27	24 Not well-tolerated when tested due to constriction of pharyngeal muscles on exspiration	Scopolamine started 1 month before death	Yes	27
11	66–70	11	Dysarthria	No	28	23	n.a.	Scopolamine lead to skin irritation. Botulinum toxine with mediocre effect.	No	n.a.
12	76–80	7	Dysarthria	No	n.a.	n.a.	n.a.	Scopolamine discussed, patient hesitant.	No	n.a.
13	80–85	5	Dysarthria + dysphagia	Yes	None	5	No indication	Scopolamine started 1 month before death	Yes, but not tolerated	7
14	50–55	15	Dysarthria	No	n.a.	n.a.	n.a.	Scopolamine currently with good effect on drooling.	No	n.a.

*NIV, non-invasive ventilation; TIV, tracheostomy invasive ventilation; n.a., not applicable; Age at onset was given as a range in order to avoid indirectly identifying data of patients*.

**Table 1B:** Demographic data and timeframe of interventions of patient cohort with progressive bulbar palsy.

	**Median (range)**	
Age at onset (years)	68.5 (53–80)	*n* = 14
Time to diagnosis (months)	10.5 (3–16)	*n* = 14
First spinal symptoms (months since onset)	24 (12–30)	*n* = 12
Gastrostomy placement (months since onset)	21 (5–27)	*n* = 11
Survival time after gastrostomy (months)	7 (0–15)	*n* = 9
NIV initiation (months since onset)	23 (16–30)	*n* = 7
Death (months since onset)	27.5 (7–37)	*n* = 10

The duration of isolated bulbar symptoms was longer than defined by Chio et al. (3), and the median time until first spinal symptoms (muscle weakness of limbs, spasticity of limbs) were documented was 24 months. One patient did not develop any spinal symptoms until death (No. 13) and two of the surviving patients still have no spinal symptoms (Nos. 12 and 14). All patients took riluzole in a standard dosage.

### Symptomatic Treatment

#### Saliva/Drooling, Thick Mucus

Sialorrhea was the most challenging symptom in all 14 patients (see Table 1A). Anticholinergic drugs (scopolamine and amitriptyline) were recommended to all patients. Initially, these showed an effect on drooling in most patients. Two patients refused medication against sialorrhea despite severe drooling due to a lack of belief in the effectiveness of medication (Nos. 7 and 12). The scopolamine patch had to be terminated due to acute cognitive alterations in one patient (No. 4). Another patient taking amitriptyline developed circulatory problems (No. 5). Four patients received injections of botulinum toxin into salivary glands (Nos. 1, 6, 8, and 11). The only patient who received radiotherapy after initial therapy with botulinum toxin did not show satisfying results with both treatments (No 8). Another patient was considered for radiotherapy (No. 6), but this was not feasible because the patient was not able to lie flat for long enough to tolerate the procedure.

The majority of patients (9 out of 14) developed thick mucus later in the disease, which did not respond well to anticholinergic drugs. In seven patients, a mechanical insufflator/exsufflator device to loosen thick mucus was introduced, but was not tolerated by three patients (Nos. 6, 8, and 13).

### Interventions

#### Gastrostomy

A total of 11 (Nos. 1–6, 8, 9, 10, 11, and 13) of the 14 patients with severe dysphagia and/or weight loss underwent placement of a feeding tube, percutaneous endoscopic gastrostomy tube (PEG). Median time from onset of diagnosis until PEG placement was 21 months (range, 5–27 months). Only one patient (No. 7) did not decide on feeding tube placement, despite several discussions on this topic and severe dysphagia and weight loss. Patient No. 10 died suddenly 4 days after PEG placement in the hospital without signs of acute infection. Median survival time after gastrostomy was 7 months (range, 0–15).

#### Non-invasive Ventilation (NIV), Invasive Ventilation

In six patients (Nos. 1, 2, 4, 5, 6, and 10), NIV was started when symptoms of nocturnal hypoventilation occurred. For patient No. 3, NIV was initiated in acute ventilatory insufficiency during bronchopulmonary infection and this patient died 1 week later. All other patients (Nos. 1, 2, 4, 5, 6, and 10) who started NIV did not tolerate this well, and the causes for NIV intolerance are described in Table 1B. Only one patient (No. 5) was able to use NIV for 3–4 h per night but did not tolerate NIV in the second half of the night due to severe dryness of the mouth. Later, this patient underwent tracheostomy and is still, to our knowledge, receiving invasive ventilation. The circumstances that led to tracheostomy could not be clarified.

One patient (No. 8) actively decided against initiating NIV. The other six patients have not (yet) developed symptoms of nocturnal hypoventilation or shown hypercapnia in arterial blood gas analysis.

#### End-of-Life Phase and Place of Death

Six out of 10 patients died in the hospital, three of them on an intensive care unit (ICU), two on a neurological ward, and one on a palliative care ward. Three patients died in hospice care. Only one patient died at home 3 weeks after discharge from a palliative care ward (see Table 2). Four patients died in the context of bronchopulmonary infection and subsequent ventilatory insufficiency (patient Nos. 3, 8, 9, and 13). The combination of excessive oral secretion and ventilatory insufficiency, without clinical signs of infection, was central in the end-of-life phase of four patients (Nos. 1, 2, 4, and 6). Patient No. 1 declined rapidly due to excessive oral secretion. She was admitted to an ICU and non-invasive ventilation was attempted, but due to collapsing pharyngeal muscles, this was not effective, and like Patient No. 3, invasive ventilation was not attempted as this was not in accordance with their expressed wishes. Patient 7 died due to severe traumatic brain injury after a fall on the stairs at home. She underwent emergency intubation, but invasive ventilation was withdrawn when the patient's will was communicated by her family. Patient 10 died 4 days after PEG tube placement without signs of acute infection, probably due to increasing hypercapnia because of postinterventional immobility. NIV had been trialed but had not been tolerated and therefore had been terminated 3 months before, when the physical decline had begun following an episode of pneumonia. Patient No. 13 died in the context of bronchopulmonary infection despite intravenous antibiotic treatment, possibly due to asphyxia. An autopsy was not performed on these two patients.

**Table 2:** Terminal phase of PBP patients.

**Patient no**.	**Place of death**	**Circumstances of dying**	**Symptom control in terminal phase**
1	ICU	Ventilatory insufficiency, excessive oral secretion. NIV non-efficient due to collapsing pharyngeal muscles, no signs of infection. No invasive ventilation according to patient‘s will.	Morphine i.v.
2	Palliative care ward	Ventilatory insuffiency and excessive oral secretion	No information
3	ICU	Progressive ventilatory insufficiency, bronchopulmonary infection treated with antibiotics 1 week prior. NIV, no invasive ventilation according to patient‘s will	Morphine, Midazolam i.v.
4	Hospice care	Ventilatory insufficiency, excessive oral secretion	Morphine p.o.
6	Hospice care	Ventilatory insufficiency and excessive oral secretion	Morphine p.o.
7	ICU	Severe traumatic brain injury after domestic stair fall 1 day before. Invasive ventilation terminated according to patient‘s will.	No information on medication used
8	Hospice care	Ventilatory insufficiency. Bronchopulmonary infection treated with antibiotics twice (8 and 6 weeks prior to death) on Neurology ward, then transfer to palliative care ward, then transfer to hospice for the last 3 weeks.	Morphine s.c.
9	At home	Ventilatory insufficiency. Bronchopulmonary infection and antibiotic therapy on neurological ward 5 weeks prior to death, then transfer to palliative care ward. Stabilization during the 2 weeks on palliative care ward and decision not to treat further infections with antibiotics. Died 3 weeks after discharge from palliative care ward due to recurrent bronchopulmonary infection.	Morphine s.c.
10	Neurology ward in hospital	Sudden death, 4 days after PEG placement. Possibly due to hypercapnia, NIV not tolerated. 4 months prior first pulmonary infection and NIV initiation	Sudden death
13	Neurology ward in hospital	Severe thick mucus accumulation, bronchopulmonary infection, 4 days of i.v. antibiotics, patient died suddenly probably due to asphyxia	Morphine p.o. against nightly cough attacks

*ICU, intensive care unit; i.v., intravenously; p.o., per os*.

Symptom control in the terminal phase was successful in all patients for whom this information could be retrieved (Nos. 1, 3, 4, 6, 8, and 9; see Table 2). Most received morphine either intravenously or subcutaneously; patient No. 3 also received midazolam intravenously. Patient No. 2 was transferred to a palliative care ward in her home town and we were not able to retrieve further information about her. Patient Nos. 4 and 6, who died in hospice care, were treated with oral morphine.

## Discussion

In our analysis of a retrospective case series of 14 patients with progressive bulbar palsy (PBP), the special needs of this patient group can be seen, together with the role of palliative care. PBP patients may stay independent in self-care and mobility but have many symptoms, and medical interventions can lead to specific challenges.

The median time of survival and female predominance was similar to the characteristics defined by others (3, 5). A median survival time after feeding tube placement of 7 months was comparable to data of a large prospective cohort study (9). Gastrostomy should be openly and early discussed with PBP patients, but studies show that gastrostomy feeding prevented weight loss in only half of ALS patients, and in those who gained weight, the clinical benefit was unclear (9). Moreover, airway secretion accumulation is a major risk factor and increases the perioperative risk by 2.6 (10). In our view, the decision for gastrostomy feeding should be based on the assessment of quality of life. Caregiver burden due to gastrostomy feeding is not high, as PBP patients remain autonomous for a longer period before paresis of the limbs may limit self-care.

Excessive oral secretion, which frequently worsens after feeding tube placement, is a major burdensome symptom in PBP patients and a challenge to manage. Therefore, symptomatic treatment of this symptom must be attempted before PEG insertion and especially during the healing process. First-line therapies are anticholinergic substances, such as scopolamine or amitriptyline. Alternatively, sublingual application of atropine eye drops or glycopyrrolate can be considered, which is available for subcutaneous or oral application (11). Botulinum toxin A injections into salivary glands is available as second-line therapy for sialorrhea (12).

PBP patients may not tolerate non-invasive ventilation (NIV) as well as ALS patients with limb phenotype, and this has been included in treatment guidelines (12). Only one of our patients tolerated NIV for more than 3–4 h. This may have been due to several aspects: excessive saliva and/or thick mucus is a major issue of distress in this cohort, which often cannot be treated with satisfying results and impairs usage of ventilation masks; muscular weakness of the pharynx leads to constriction mainly in exsufflation which cannot be technically compensated. The risks and complications of NIV should be openly discussed with patients before initiation of NIV to prevent frustration. For clinicians, the anticipation of potential obstacles when initiating NIV in ALS patients is very important in order to ensure that NIV is able to provide effective help and is acceptable to the patient. In addition to optimizing secretion management, Baxter et al. (13) recommend the following when initiating NIV: easily accessible in-person advice, the use of humidifiers and alternative mask interfaces, and discussing the potential benefits of NIV in detail with patients.

In our experience, only very few patients with progressive bulbar palsy decide to undergo tracheostomy if this aspect is discussed as part of ACP in the course of the disease. Only one of our patients underwent tracheostomy and is still, to our knowledge, under mechanical ventilation. This patient, however, has been lost to follow-up and we do not know under which circumstances tracheostomy was performed. She now lives in a specialized respiratory care facility.

Recognizing the end-of-life phase in PBP patients can be challenging, as they often maintain a relatively high functional status with mild to moderate limb paresis. However, PBP patients deteriorate quickly, and to avoid unwanted hospitalizations and to ensure adequate palliative care at the end of life, this phase has to be identified in a timely manner. The implementation of specific triggers predicting the end-of-life phase can help to increase palliative care input and prepare patients and caregivers. Studies to assess the value of triggers for palliative care involvement in neurological patients have shown that the number of triggers increases rapidly in the last 6 months of life (14, 15). Hussein et al. identified key factors that seemed to influence the deterioration of neurological patients in the last 6 months of life in particular: decline in physical function, weight loss and respiratory symptoms, recurrent infections and cognitive impairment, and aspiration (15). Triggers that indicate the end-of-life phase in ALS in general have been established by expert consensus and include swallowing problems, recurrent pulmonary infection, marked decline in functional status, cognitive difficulties, weight loss, and significant complex symptoms (2, 16). In PBP, however, some of these symptoms may have been seen earlier in the disease course. In this patient group, rapid decline began in the context of bronchopulmonary infection in at least five patients, and excessive oral secretion could be recognized in all these patients prior to the pneumonia. Therefore, we consider the time of first pulmonary infection to be a crucial point toward the end-of-life phase in patients with PBP, as was seen in early studies (14).

Communication with patients with PBP and their caregivers should take these issues into account, and we wish to emphasize that ACP conversations with patients and families are crucial—as it is with all ALS patients (17). In particular, pseudohypersalivation and associated bronchopulmonary infection as risk factors have to be discussed thoroughly with patients and relatives. The difficulties, or even inability, to communicate verbally as the disease progresses have to be taken into account. In an acute care setting, many patients will not be able to participate in end-of-life discussions to the extent that they would like to. Risks and benefits of life-sustaining interventions, such as emergency intubation, tracheostomy, and gastrostomy tube should be discussed early and regularly.

As we have shown in our patient group, PBP is often associated with a rapid and often not foreseeable decline, and therefore, it can be challenging to care for these patients at his or her own home. Only 1 of the 10 deceased PBP patients died at home, much less than expected according to older data, where ~50% of German ALS patients died at home (18). Moreover, five patients died in an acute setting on a Neurology ward or ICU. High symptom burden at the end of life and rapid decline seem to be more pronounced in this ALS subgroup. Therefore, this group of patients should be involved in ACP early and remain in frequent contact with specialized nurses and physicians (19). It is important to inform patients and relatives and prepare them for an increase in symptom burden, and there may be effective treatment. Anticipatory prescription of on-demand medication, such as morphine for the treatment of dyspnea (20), and early involvement of palliative care services can avert emergency hospitalization and enable dying at home.

A limitation of our study is the small number of patients, the retrospective design, and incomplete information for some patients. Furthermore, we do not have any pathological or genetic data of our patients.

It is important to recognize that patients with PBP form a subgroup of ALS with distinct features. Due to short survival time and possible impairment of decision-making capacity, early and accurate information of patients and caregivers are highly important. Possible rapid deterioration at the end of life should also be kept in mind as an additional challenge in palliative care.

## Data Availability Statement

The original contributions presented in the study are included in the article/supplementary material, further inquiries can be directed to the corresponding author/s.

## Ethics Statement

Ethical review and approval was not required for the study on human participants in accordance with the local legislation and institutional requirements. Written informed consent for participation was not required for this study in accordance with the national legislation and the institutional requirements.

## Author Contributions

SB collected and analyzed patient data and wrote the manuscript. CW, AE-R, KL, PP, and SL participated in discussions on results and reviewed the manuscript. All authors contributed to the article and approved the submitted version.

## Conflict of Interest

The authors declare that the research was conducted in the absence of any commercial or financial relationships that could be construed as a potential conflict of interest. The handling editor declared a past co-authorship with one of the authors, SL.

## Publisher's Note

All claims expressed in this article are solely those of the authors and do not necessarily represent those of their affiliated organizations, or those of the publisher, the editors and the reviewers. Any product that may be evaluated in this article, or claim that may be made by its manufacturer, is not guaranteed or endorsed by the publisher.
